# Early Growth Response 1‐Associated Epithelial Autophagy‐Related Changes in Allergic Airway Inflammation

**DOI:** 10.1002/pdi3.70075

**Published:** 2026-09-25

**Authors:** Xinyang Wang, Jie Ren, Yan Zhao, Zhengxiu Luo

**Affiliations:** ^1^ Department of Respiratory Medicine Children's Hospital of Chongqing Medical University, National Clinical Research Center for Children and Adolescents' Health and Diseases, Ministry of Education Key Laboratory of Child Development and Disorders, Chongqing Key Laboratory of Child Rare Diseases in Infection and Immunity, Chongqing Municipal Health Commission Key Laboratory of Children's Vital Organ Development and Diseases Chongqing China

## Abstract

Airway epithelial cells contribute critically to the initiation of allergic airway inflammation via the release of alarmins such as interleukin‐33 (IL‐33). Nevertheless, the intracellular regulatory networks governing epithelial alarmin release remain inadequately defined. Although autophagy has been implicated in epithelial stress adaptation and the transcription factor early growth response 1 (*EGR1*) is promptly induced upon allergen challenge, it remains uncertain whether *EGR1* contributes to IL‐33 release as a representative indicator of epithelial activation through autophagy‐associated processes. In this study, human bronchial epithelial BEAS‐2B cells were exposed to house dust mite (HDM) extract. *EGR1* expression was manipulated using gain‐ and loss‐of‐function strategies. Autophagy‐associated alterations were evaluated by monitoring LC3B, p62, and PI3K‐III (Vps34) levels, together with immunofluorescence microscopy. IL‐33 release into culture supernatants was quantified by ELISA as a functional readout of epithelial activation. Pharmacological interference with autophagy was employed to examine functional engagement. An HDM‐driven murine model was utilized to assess in vivo correlates. Publicly accessible transcriptomic datasets were interrogated in an exploratory manner to characterize epithelial stress signatures linked to this axis. HDM treatment upregulated *EGR1* in airway epithelial cells, concomitant with autophagy‐associated alterations and enhanced IL‐33 release. Forced *EGR1* expression coincided with elevated LC3B‐II levels, diminished p62 accumulation, and increased PI3K‐III abundance, whereas *EGR1* silencing produced reciprocal effects. Pharmacological suppression of autophagy attenuated IL‐33 release and inhibition of Class III PI3K blunted the *EGR1* overexpression‐driven increase in IL‐33 release. In HDM‐challenged mice, colocalization of EGR1 and LC3B was observed in lung tissue sections. Exploratory transcriptomic profiling further suggested that allergen provocation may elicit a coordinated epithelial stress response characterized by inflammatory and metabolic pathway enrichment. The results show that *EGR1* is associated with epithelial autophagy‐related alterations under allergic stimulation, and these changes are accompanied by augmented release of the alarmin IL‐33. These observations support a conceptual framework wherein *EGR1*‐linked autophagy‐associated processes may contribute to epithelial stress responses during early inflammatory events in allergic airway disease. Elucidation of the precise molecular underpinnings and causal relationships awaits further investigation.

## Graphical abstract

House dust mite (HDM) exposure induces early growth response 1 (*EGR1*) expression in airway epithelial cells and is accompanied by autophagy‐associated alterations, including increased LC3B‐II and Vps34 levels and decreased p62 abundance. Manipulation of *EGR1* expression modulates these autophagy‐related changes, whereas pharmacological inhibition of Class III PI3K attenuates epithelial activation. In vivo, HDM exposure further demonstrates colocalization of EGR1 and LC3B in lung tissue. Exploratory transcriptomic analysis suggests that these responses may occur within a broader epithelial stress and inflammatory program during allergic airway inflammation.

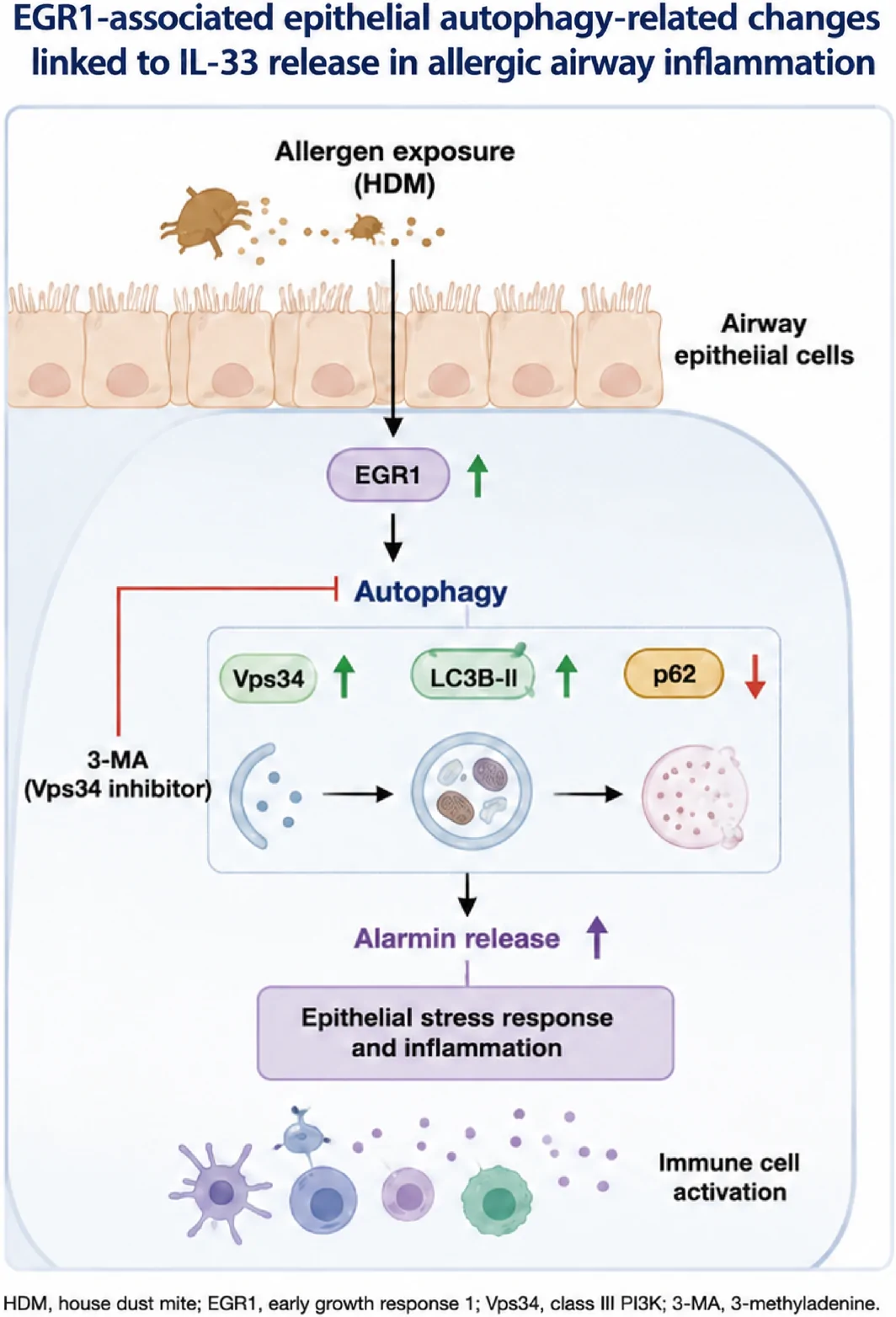

## 1 Introduction

Asthma represents one of the most prevalent chronic respiratory disorders in pediatric populations and is hallmarked by persistent airway inflammation, airway hyperresponsiveness, and recurrent wheezing episodes [1, 2]. It exacts a considerable toll on the affected individuals and the healthcare infrastructure. Mounting evidence indicates that airway epithelial cells function not merely as a passive structural barrier but also as primary sentinels perceive environmental cues [3, 4, 5]. In response to allergen exposure, epithelial cells rapidly discharge alarmins—including interleukin‐33 (IL‐33), thymic stromal lymphopoietin (TSLP), and IL‐25—which subsequently initiate and propagate Type 2 immune cascades, culminating in eosinophilic inflammation, mucus overproduction, and airway wall remodeling [6, 7, 8, 9, 10]. Despite their acknowledged importance, the intracellular mechanisms that dictate epithelial alarmin release remain incompletely elucidated.

Autophagy is an evolutionarily conserved intracellular degradative pathway that preserves cellular homeostasis through the clearance of damaged organelles and aberrant protein aggregates [11, 12]. Beyond its canonical catabolic function, emerging evidence has revealed that autophagy additionally participates in stress adaptation, modulation of inflammatory responses, and unconventional protein export [13, 14, 15, 16]. Within the respiratory tract, autophagy has been linked to epithelial injury, airway inflammation, and phenotypic heterogeneity of disease [13, 14, 15, 16]. In the setting of allergic airway inflammation, autophagy‐related events may shape epithelial responsiveness to environmental stressors and could influence the handling of inflammatory mediators such as IL‐33 [13, 14]. However, whether autophagy directly participates in IL‐33 release as a representative indicator of epithelial activation, and how this process is governed at upstream checkpoints, remain open questions.

Class III phosphatidylinositol 3‐kinase (PI3K‐III, also termed Vps34/PIK3C3) functions as a pivotal initiator of autophagy, catalyzing the generation of phosphatidylinositol 3‐phosphate (PI3P) and facilitating autophagosome nucleation [11, 12]. As a central conduit linking upstream stress inputs to downstream autophagic execution, PI3K‐III offers a mechanistic vantage point that extends beyond descriptive assessment of surrogate markers such as LC3B and p62. Early growth response 1 (*EGR1*) constitutes an immediate‐early transcription factor that is swiftly induced by inflammatory mediators, oxidative stress, and environmental perturbations and has been implicated in orchestrating gene expression programs pertinent to airway inflammation [17, 18, 19]. Earlier studies have noted an association between *EGR1* and epithelial inflammatory responses in Type 2 airway inflammation [20]. Notably, recent work from our own group established that EGR1 can directly govern the transcription of certain epithelial‐derived cytokines [20]. Although the transcriptional regulation of alarmins by EGR1 has been recognized, whether EGR1 also coordinates the upstream cellular stress and autophagy machinery in airway epithelial cells remains poorly defined.

In this study, we employed both in vitro and in vivo models of house dust mite (HDM)‐elicited allergic airway inflammation to delineate the expression profiles of *EGR1* and autophagy‐associated markers. By combining gain‐ and loss‐of‐function manipulation of *EGR1* with pharmacological modulation of autophagy, we sought to determine whether *EGR1* correlates with PI3K‐III‐involved autophagy‐related processes and to characterize the downstream functional consequences of this axis on IL‐33 release as a representative indicator of epithelial activation. Furthermore, we performed an exploratory analysis of publicly deposited transcriptomic data to evaluate whether this pathway may form part of a broader epithelial stress response triggered by allergen challenge.

## 2 Methods

### 2.1 Animal Model of HDM‐Induced Allergic Airway Inflammation

Female C57BL/6JGpt mice (8 weeks old) were purchased from Chongqing Jilihui Biological Co. Ltd., China, and maintained under a 12/12‐hour dark/light cycle in a controlled environment at approximately 23°C with 40%–50% humidity. Mice were randomly assigned to control and asthma groups (*n* = 4 per group). In the asthma group, each mouse received intranasal administration of 20 μg/40 μL HDM (*Dermatophagoides pteronyssinus*, Greer Laboratories, USA) during the sensitization phase (days 0 and 14) and the challenge phase (days 21, 23, 25, 27, and 29). Control animals received an equivalent volume of 0.9% sterile saline at the same time points. Pulmonary function testing was performed on day 30. Mice were euthanized on day 31, and lung tissue samples were collected. All animal procedures were approved by the Institutional Animal Care and Use Committee, Children's Hospital of Chongqing Medical University (Approval No. CHCMU‐IACUC20230804005).

### 2.2 Cell Culture and HDM Stimulation

The human bronchial epithelial cell line BEAS‐2B was cultured in DMEM (Gibco, Waltham, MA, USA) supplemented with 10% fetal bovine serum (Gibco, Waltham, MA, USA) and 1% penicillin‐streptomycin solution (NCM Biotech, Suzhou, Jiangsu, China) at 37°C in a humidified incubator with 5% CO_2_. To establish an in vitro asthma model, BEAS‐2B cells were incubated with or without HDM extract (30 μg/mL) for 3 hours.

### 2.3 *EGR1* Overexpression and Knockdown

For gain‐ and loss‐of‐function experiments, BEAS‐2B cells were seeded in 12‐well plates at a density of 1.5 × 10^5^ cells per well. When cells reached 60%–70% confluence, transient transfections were performed using Lipofectamine 2000 (Invitrogen, USA) according to the manufacturer's standard protocol. For *EGR1* knockdown, cells were transfected with small interfering RNA (siRNA) targeting *EGR1* (Sangon Biotech, Shanghai, China) at a final concentration of 50 nmol/L with 2 μL Lipofectamine 2000 per well. The siRNA sequences were as follows: sense 5′‐GCAGCAGCAGCACCUUCAA TT‐3′, and antisense 5′‐UUGAAGGUGCUGCUGCUGC TT‐3′. For *EGR1* overexpression, cells were transfected with a 1‐μg pcDNA3.1(+) plasmid encoding full‐length human *EGR1* (GeneCreate, Wuhan, Hubei, China) with 2 μL Lipofectamine 2000 per well. After 48 hours of transfection, cells were treated with or without HDM (30 μg/mL) for an additional 3 hours. Transfection efficiency was verified by quantitative PCR and Western blot analysis. All experiments were performed in at least three independent replicates.

### 2.4 Pharmacological Inhibition of Autophagy

For autophagy inhibition, BEAS‐2B cells were incubated with 10 mmol/L 3‐methyladenine (3‐MA; MedChemExpress, Monmouth Junction, NJ, USA) for 6 hours prior to downstream analyses.

### 2.5 Quantitative Real‐Time Polymerase Chain Reaction (PCR)

Total RNA was extracted from cells and tissues using total RNA extraction reagent (BioFlux, Beijing, China). Extracted RNA was reverse‐transcribed into cDNA using the Evo M‐MLV RT reaction mix kit (Accurate Biology, Changsha, Hunan, China) following the manufacturer's instructions. Quantitative real‐time PCR (RT‐qPCR) was performed using the SYBR Green Pro Taq HS Premixed qPCR Kit (Accurate Biology, Changsha, Hunan, China) on a Bio‐Rad CFX PCR system (Bio‐Rad, Hercules, CA, USA). Each reaction (total volume 10 μL) contained 5 μL SYBR Green Pro Taq HS Premix, 0.2 μL of each specific primer, 2.6 μL RNase‐free water, and 2 μL cDNA. Primer sequences are provided in Table 1. The PCR protocol consisted of 40 cycles at 95°C for 10 seconds and 63.3°C for 30 seconds. Relative EGR1 expression was calculated using the 2^−^^ΔΔCt^ method with GAPDH as the internal reference.

**TABLE 1:** Primer sequences (H: Human; M: Mouse).

Gene	Sequence (5′→3′)
H‐EGR1‐forward H‐EGR1‐reverse	CCGCAGAGTCTTTTCCTGAC AGCGGCCAGTATAGGTGATG
H‐GAPDH‐forward H‐GAPDH‐reverse	GCACCGTCAAGGCTGAGAAC TGGTGAAGACGCCAGTGGA
M‐EGR1‐forward M‐EGR1‐reverse	TGACCAATCCTCCGACCTCT AGATGGGACTGCTGTCGTTG
M‐GAPDH‐forward M‐GAPDH‐reverse	CATCACTGCCACCCAGAAGACTG ATGCCAGTGAGCTTCCCGTTCAG

### 2.6 Western Blot Analysis

Cells were harvested, and denatured protein samples were resolved on 10% SDS‐polyacrylamide gels, electrophoresed, and wet‐transferred onto polyvinylidene difluoride (PVDF) membranes. Membranes were blocked with 5% bovine serum albumin at room temperature and incubated with primary antibodies overnight at 4°C, followed by incubation with horseradish peroxidase‐conjugated secondary antibodies for 1 hour at room temperature. Protein bands were visualized using ECL reagent and a Bio‐Rad gel imaging system (Bio‐Rad, Hercules, CA, USA). Primary antibodies used in this study included the following: anti‐EGR1 rabbit monoclonal antibody (1:800 dilution; Cell Signaling Technology, Danvers, MA, USA), anti‐SQSTM1/p62 rabbit monoclonal antibody (1:800 dilution; ZenBio, Chengdu, Sichuan, China), anti‐LC3B rabbit monoclonal antibody (1:800 dilution; ABclonal, Wuhan, Hubei, China), anti‐PI3 kinase Class 3 (Vps34) rabbit monoclonal antibody (1:800 dilution; Upingbio, Wuhan, Hubei, China), and anti‐GAPDH HRP‐conjugated monoclonal antibody (1:10,000 dilution; ABclonal, Wuhan, Hubei, China). The secondary antibody was anti‐rabbit IgG HRP‐conjugated antibody (1:10,000 dilution; ZenBio, Chengdu, Sichuan, China). The details are summarized in Table 2.

**TABLE 2:** Antibodies used in this study.

Name	Supplier	Cat no.	Clone no.
EGR1 (44D5) rabbit monoclonal antibody	Cell Signaling Technology (CST)	4154	44D5
PI3 kinase Class 3 polyclonal antibody	Upingbio	YP‐mAb‐10588	N/A
SQSTM1/p62 rabbit mAb	ZenBio	R380612	R05‐5P‐6
LC3B rabbit mAb	ABclonal	A19665	ARC0144
HRP‐conjugated GAPDH rabbit mAb	ABclonal	AC054	ARC50888‐HRP

### 2.7 Immunofluorescence Staining

For tissue immunofluorescence, paraffin‐embedded lung sections (4 μm thickness) were deparaffinized and rehydrated. Antigen retrieval was performed, followed by incubation with anti‐EGR1 antibody (1:200 dilution; Cell Signaling Technology, Danvers, MA, USA) overnight at 4°C. The following day, sections were incubated with fluorophore‐conjugated secondary antibody (donkey anti‐rabbit IgG H+L, Alexa Fluor 488, 1:500 dilution; Thermo Fisher Scientific, Waltham, MA, USA) and mounted with antifade mounting medium containing DAPI (Beyotime, Shanghai, China). For cell immunofluorescence, BEAS‐2B cells were fixed with 4% paraformaldehyde, permeabilized, and incubated with primary antibodies against LC3B (1:200 dilution; ABclonal, Wuhan, Hubei, China) or EGR1 (1:200 dilution; Cell Signaling Technology, Danvers, MA, USA), followed by fluorophore‐conjugated secondary antibodies. Nuclei were counterstained with DAPI. Images were acquired using a confocal microscope (Nikon C2+, Nikon Corporation, Tokyo, Japan) at ×20 or ×60 magnification, and LC3B puncta were quantified.

### 2.8 Enzyme‐Linked Immunosorbent Assay (ELISA)

Cell culture supernatants were collected, and IL‐33 protein levels were measured using a commercial ELISA kit (MeiMian, Yancheng, Jiangsu, China) according to the manufacturer's instructions. IL‐33 release into the supernatant was employed as a surrogate functional readout of epithelial activation.

### 2.9 Exploratory Transcriptomic Analysis

Public RNA‐seq data were obtained from the Gene Expression Omnibus (GEO) database under accession number GSE243680, which includes human bronchial epithelial BEAS‐2B cells stimulated with HDM extracts and corresponding mock controls at 4‐ and 24‐hour time points. Raw count data generated on the Illumina NovaSeq 6000 platform were used for downstream analysis. Genes with low expression levels (total counts ≤ 10 across all samples) were filtered out. Count data were transformed using the function log_2_ (count + 1) for exploratory and visualization purposes. Given the limited number of control samples in the dataset, formal statistical inference based on variance estimation was not performed. Instead, a descriptive differential expression strategy based on log_2_ fold change (logFC) ranking was employed to visualize global transcriptional trends. Functional enrichment analysis was performed using the Database for Annotation, Visualization, and Integrated Discovery (DAVID, version 6.8) in an exploratory capacity to identify pathways potentially associated with HDM‐induced epithelial responses. All analyses were performed using R (version 4.5.2). Results from this analysis should be interpreted as hypothesis‐generating rather than definitive mechanistic evidence.

### 2.10 Statistical Analysis

Statistical analyses were performed using GraphPad Prism 10 (GraphPad Software, San Diego, CA, USA). Data are presented as mean ± standard deviation (SD). The unpaired Student's t‐test, one‐way ANOVA, or repeated‐measures ANOVA were used as appropriate for each set of experimental conditions to evaluate differences between groups. Normality was assessed prior to parametric testing, and post‐hoc comparisons were performed where applicable. Differences were considered statistically significant at *p* < 0.05. Given the descriptive and associative nature of the study, no causal inference regarding IL‐33 secretion mechanisms is implied by the statistical comparisons.

## 3 Results

### 3.1 HDM Stimulation Elevates EGR1 Expression and Induces Autophagy‐Associated Alterations in Airway Epithelial Cells

**FIGURE 1:**
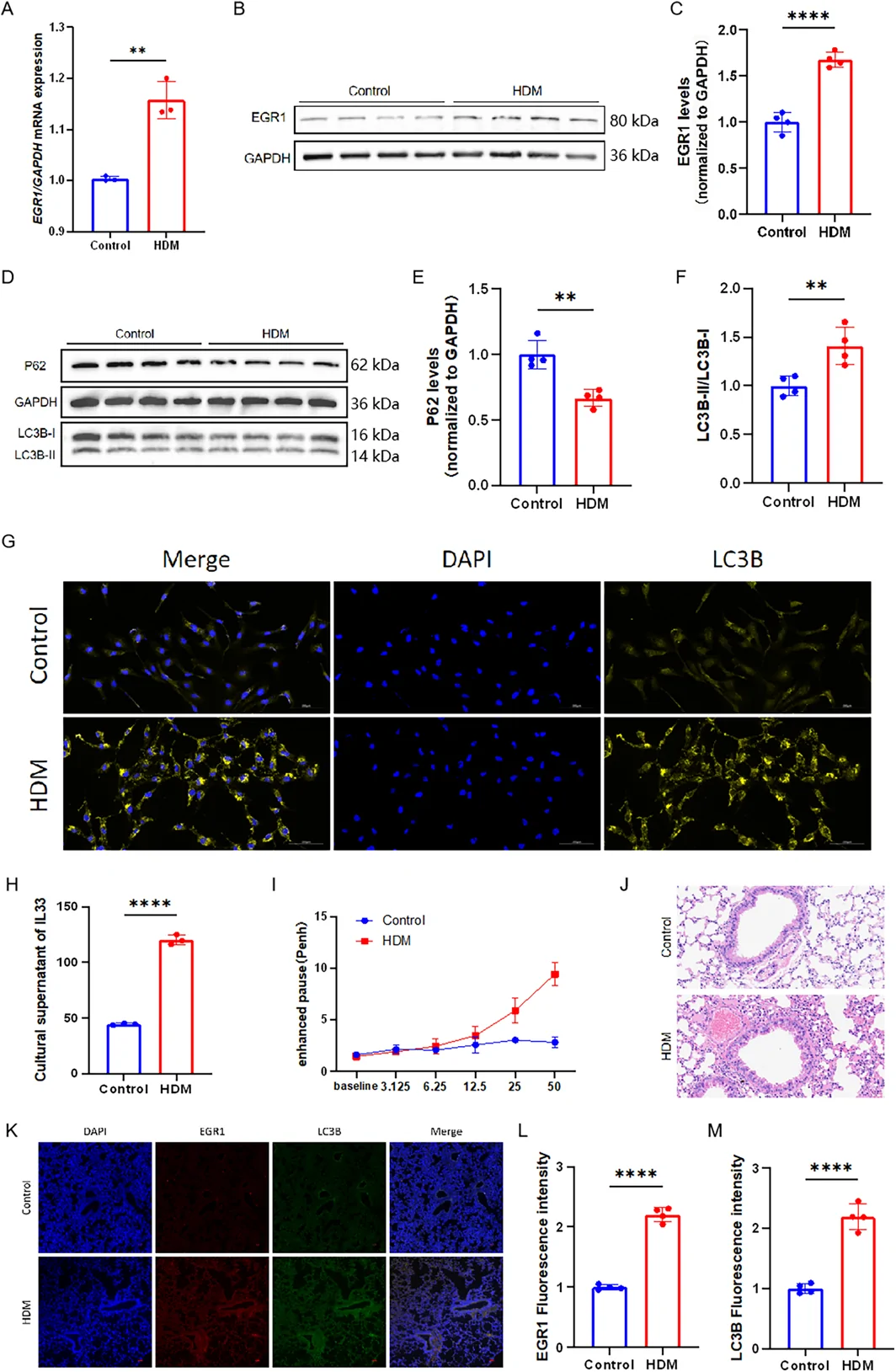
House dust mite (HDM) stimulation induces early growth response 1 (EGR1) expression and autophagy‐associated changes in airway epithelial cells. (A–C) *EGR1* mRNA and protein levels in BEAS‐2B cells stimulated with HDM extract (30 μg/mL, 3 hours) or vehicle control, assessed by quantitative polymerase chain reaction (qPCR) and Western blot. (D–F) Western blot analysis of LC3B and p62 protein levels. The LC3B‐II/I ratio was quantified by densitometry. (G) Representative immunofluorescence images of LC3B puncta (green) in BEAS‐2B cells. Nuclei were counterstained with DAPI (blue). Scale bar, 20 μm. (H) IL‐33 release into culture supernatants measured by ELISA. (I) Airway resistance in response to increasing concentrations of methacholine in HDM‐challenged mice and controls. Data were analyzed using repeated‐measures ANOVA. (J) Representative hematoxylin and eosin‐stained lung tissue sections. Scale bar, 100 μm. (K–M) Immunofluorescence detection of EGR1 (red) and LC3B (green) in lung tissue sections. Nuclei were stained with DAPI (blue). Fluorescence intensity was quantified and normalized to the control. Data are presented as mean ± standard deviation. For in vitro experiments: (A, G) *n* = 3 independent experiments; (B–F) *n* = 4 independent experiments. For in vivo experiments: *n* = 4 mice per group. Statistical significance was determined by an unpaired Student's *t*‐test unless otherwise indicated. *p < 0.05, **p < 0.01, ***p < 0.001, ****p < 0.0001. ns, not significant.

To probe *EGR1* dynamics and autophagy‐associated changes after allergen provocation, we conducted in vitro experiments with BEAS‐2B cells alongside in vivo studies in a murine asthma model. As a functional correlate, IL‐33 release into culture supernatants was also assessed. In vitro, BEAS‐2B cells were stimulated with HDM extract (30 μg/mL) for 3 hours to recapitulate an allergic epithelial milieu. *EGR1* transcript levels and EGR1 protein levels were substantially increased in the HDM‐exposed group (Figure 1A–C). The ratio of the autophagic marker LC3B‐II to LC3B‐I rose markedly, whereas p62 protein abundance declined significantly (Figure 1D–F). Immunofluorescence detection of LC3B disclosed an accumulation of punctate cytoplasmic signals (Figure 1G). IL‐33 release into culture supernatants was appreciably augmented (Figure 1H). Collectively, these observations indicate that HDM challenge is accompanied by *EGR1* upregulation, autophagy‐associated changes, and enhanced IL‐33 release in vitro.

In vivo, an asthmatic phenotype was elicited in mice through HDM exposure. Airway resistance in the asthmatic cohort was substantially greater than that in control animals (Figure 1I). Histopathological features of asthmatic lungs included thickening of bronchial walls, submucosal inflammatory infiltrates, interstitial inflammatory cell accumulation, and widening of interstitial spaces (Figure 1J). Immunofluorescence intensities for both EGR1 and LC3B were significantly elevated in the asthmatic group relative to controls (Figure 1K–M), corroborating enhanced *EGR1* expression and autophagy‐associated alterations within the asthmatic lung.

### 3.2 Manipulation of EGR1 is Paralleled by Changes in Autophagy‐Related Markers and IL‐33 Release as a Representative Indicator of Epithelial Activation

**FIGURE 2:**
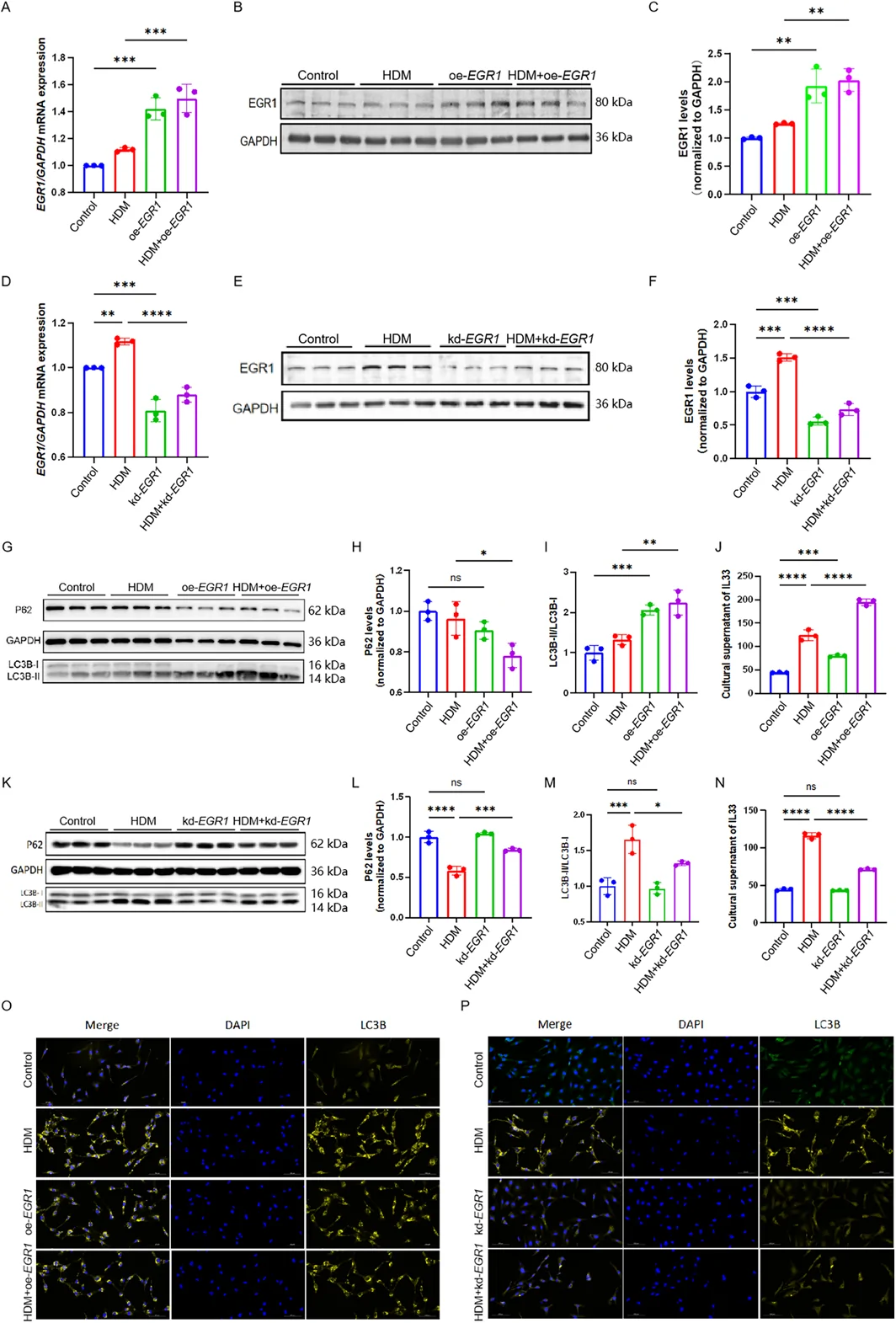
Validation of early growth indicaton 1 (*EGR1*) overexpression and knockdown efficiency, and the effects on autophagy‐related proteins and IL‐33 release in house dust mite (HDM)‐stimulated BEAS‐2B cells. (A–C) *EGR1* mRNA and EGR1 protein levels in cells transfected with the EGR1 overexpression plasmid (oe‐EGR1) or empty vector, assessed by quantitative polymerase chain reaction (qPCR) and Western blot. (D–F) *EGR1* mRNA and ERG1 protein levels in cells transfected with EGR1‐targeting siRNA (si‐EGR1) or scrambled control, assessed by qPCR and Western blot. (G–I) Western blot analysis of LC3B and p62 in oe‐EGR1‐transfected cells, followed by HDM stimulation (30 μg/mL, 3 h). The LC3B‐II/I ratio and p62 levels were quantified by densitometry. (J) IL‐33 release into culture supernatants under the same conditions, measured by ELISA. (K–M) Western blot analysis of LC3B and p62 in si‐EGR1‐transfected cells with HDM stimulation. (N) Corresponding IL‐33 release measured by ELISA. (O–P) Immunofluorescence detection of LC3B puncta (green) in oe‐EGR1 and si‐EGR1 cells stimulated with HDM. Nuclei were stained with DAPI (blue). Scale bar, 20 μm. All data are presented as mean ± standard deviation (*n* = 3 independent experiments). Statistical significance for panels (A–P) was determined by one‐way ANOVA followed by appropriate post‐hoc tests. *p < 0.05, **p < 0.01, ***p < 0.001, ****p < 0.0001. ns, not significant.

To experimentally test whether *EGR1* is associated with changes in autophagic flux and IL‐33 release as a representative indicator of epithelial activation in the context of HDM stimulation, we undertook plasmid‐mediated overexpression *EGR1* (oe‐*EGR1*) and siRNA‐mediated silencing *EGR1* (si‐*EGR1*) in vitro. *EGR1* overexpression and knockdown efficiency were validated by qPCR and western blot (Figure 2A–F). The data revealed that oe‐*EGR1* was accompanied by enhanced autophagy‐associated events in airway epithelial cells: the LC3B‐II/LC3B‐I ratio increased, p62 protein content diminished (Figure 2G–I), and IL‐33 release into the supernatant was amplified (Figure 2J). Conversely, si‐*EGR1* dampened autophagy‐related readouts and mitigated HDM‐induced effects: the LC3B‐II/LC3B‐I ratio declined, p62 levels rebounded (Figure 2K–M), and IL‐33 release was curtailed (Figure 2N). In addition, LC3B immunofluorescence displayed heightened punctate cytoplasmic staining in oe‐*EGR1* cells (Figure 2O), but reduced punctate staining in si‐*EGR1* cells (Figure 2P). These findings imply that, under HDM‐stimulated conditions, *EGR1* expression correlates with both epithelial autophagy‐associated processes and IL‐33 release as a representative indicator of epithelial activation.

### 3.3 Pharmacological Inhibition of Autophagy Attenuates *EGR1*‐Linked IL‐33 Release as a Representative Indicator of Epithelial Activation

**FIGURE 3:**
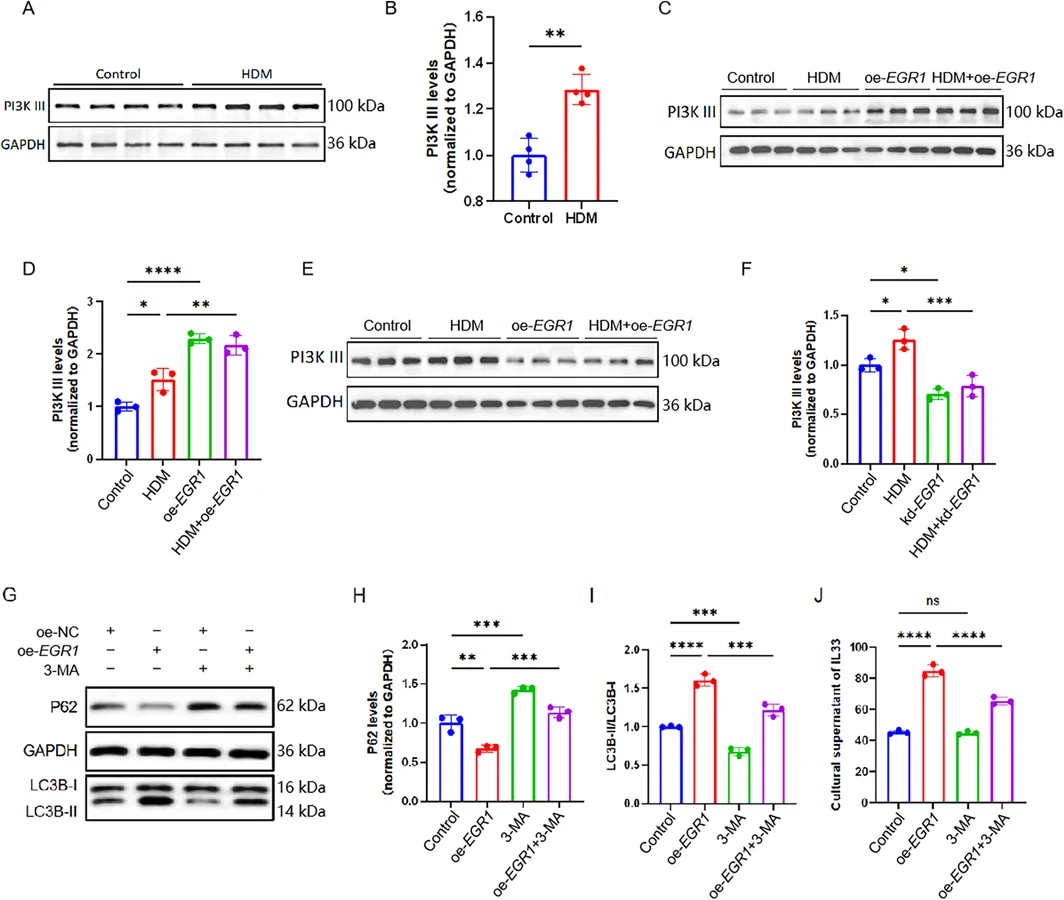
PI3K‐III (Vps34) protein levels in response to house dust mite (HDM) and early growth response 1 (EGR1) manipulation, and the impact of 3‐methyladenine (3‐MA) on autophagy and IL‐33 release in EGR1‐overexpressing cells. (A–B) Western blot analysis and quantification of Vps34 protein levels in BEAS‐2B cells stimulated with HDM extract (30 μg/mL, 3 h) or vehicle control. (C–D) Western blot analysis of Vps34 protein levels in cells transfected with oe‐EGR1 or empty vector, with or without HDM stimulation. (E–F) Western blot analysis of Vps34 protein levels in cells transfected with si‐EGR1 or scrambled control, with or without HDM stimulation. (G–I) Western blot analysis of LC3B and p62 in oe‐EGR1‐transfected cells treated with or without Class III PI3K inhibitor 3‐methyladenine (3‐MA, 10 mmol/L, 6 h). (J) IL‐33 release in the same experimental setting, measured by ELISA. All data are presented as mean ± standard deviation (*n* = 3 independent experiments). For panels (A–B), statistical significance was determined by an unpaired Student's *t*‐test. For panels (C–J), one‐way ANOVA followed by appropriate post‐hoc tests was used. *p < 0.05, **p < 0.01, ***p < 0.001, ****p < 0.0001. ns, not significant.

Given the cardinal role of Class III PI3K in autophagy initiation, we further assessed the protein abundance of its catalytic subunit Vps34 (PIK3C3) to probe the molecular interface between *EGR1* and the autophagy initiation apparatus. Vps34 (PIK3C3) levels fluctuated in a manner concordant with alterations in *EGR1* expression and autophagic activity (Figure 3A–F). These observations indicate that Class III PI3K may constitute a critical nexus linking *EGR1* and autophagy‐associated changes.

To further substantiate the functional connection between *EGR1*, autophagy‐associated processes, and IL‐33 release as a representative indicator of epithelial activation, we combined *EGR1* overexpression with pharmacological inhibition of Class III PI3K using 3‐MA in vitro. *EGR1* overexpression was accompanied by autophagy‐associated changes in airway epithelial cells (evidenced by an increased LC3B‐II/LC3B‐I ratio and decreased p62 protein) and augmented IL‐33 release, whereas cotreatment with 3‐MA attenuated these inductive effects (Figure 3G–J). These data reinforce the notion that *EGR1* is associated with epithelial autophagy‐related events and that these events are linked to IL‐33 release as a representative indicator of epithelial activation.

### 3.4 Exploratory Transcriptomic Profiling Suggests a Broader Epithelial Stress Response

**FIGURE 4:**
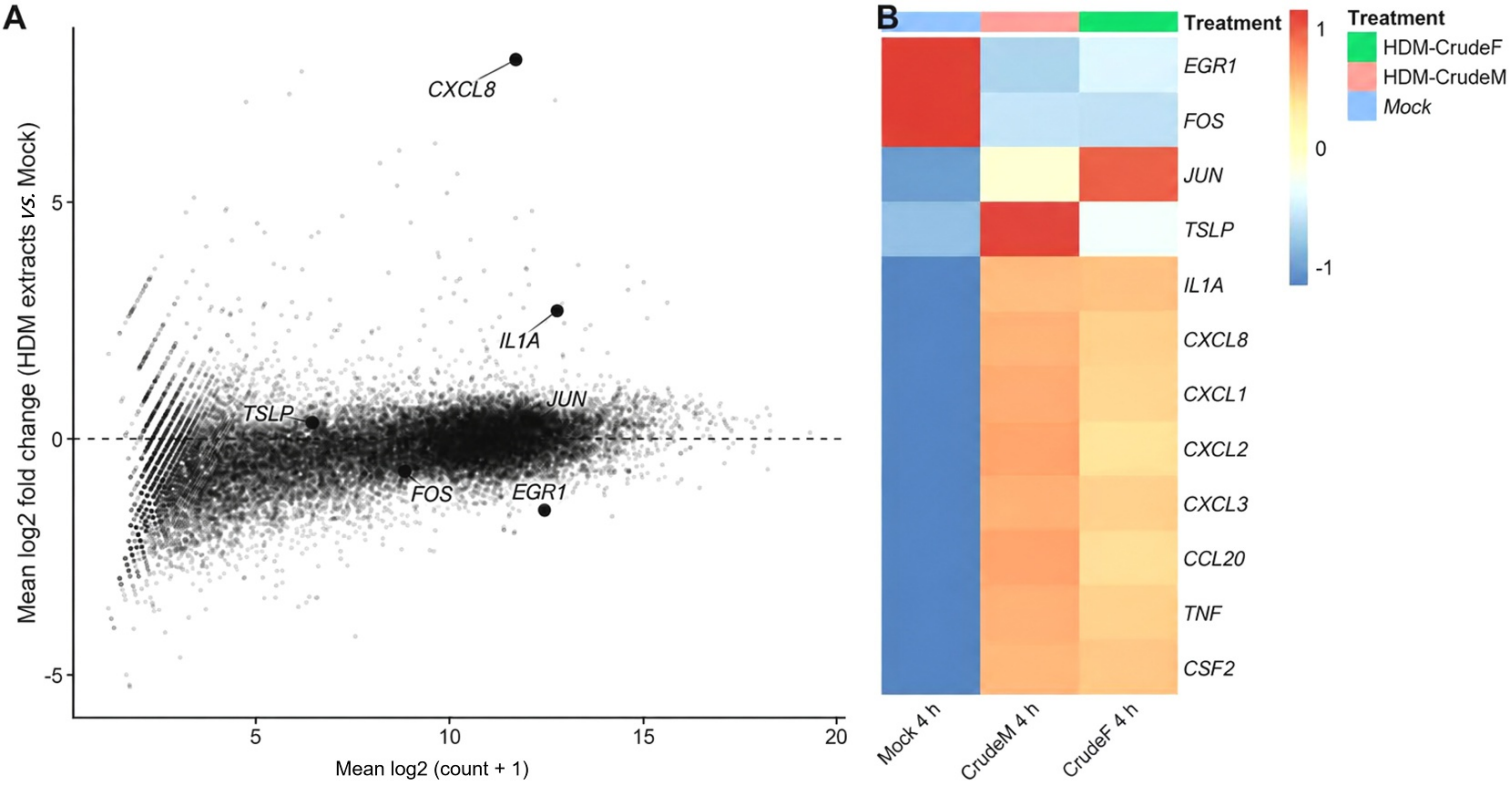
Exploratory transcriptomic profiling of house dust mite (HDM)‑stimulated airway epithelial cells. (A) Descriptive gene‑level transcriptional changes in BEAS‑2B cells following HDM stimulation, showing log_2_ fold changes of selected epithelial stress and inflammatory‑response genes. (B) Heatmap of row‑standardized expression of representative genes across mock‑ and HDM‑treated samples. All analyses are exploratory due to limited control replication, and no formal gene‑level statistical inference was applied.

**FIGURE 5:**
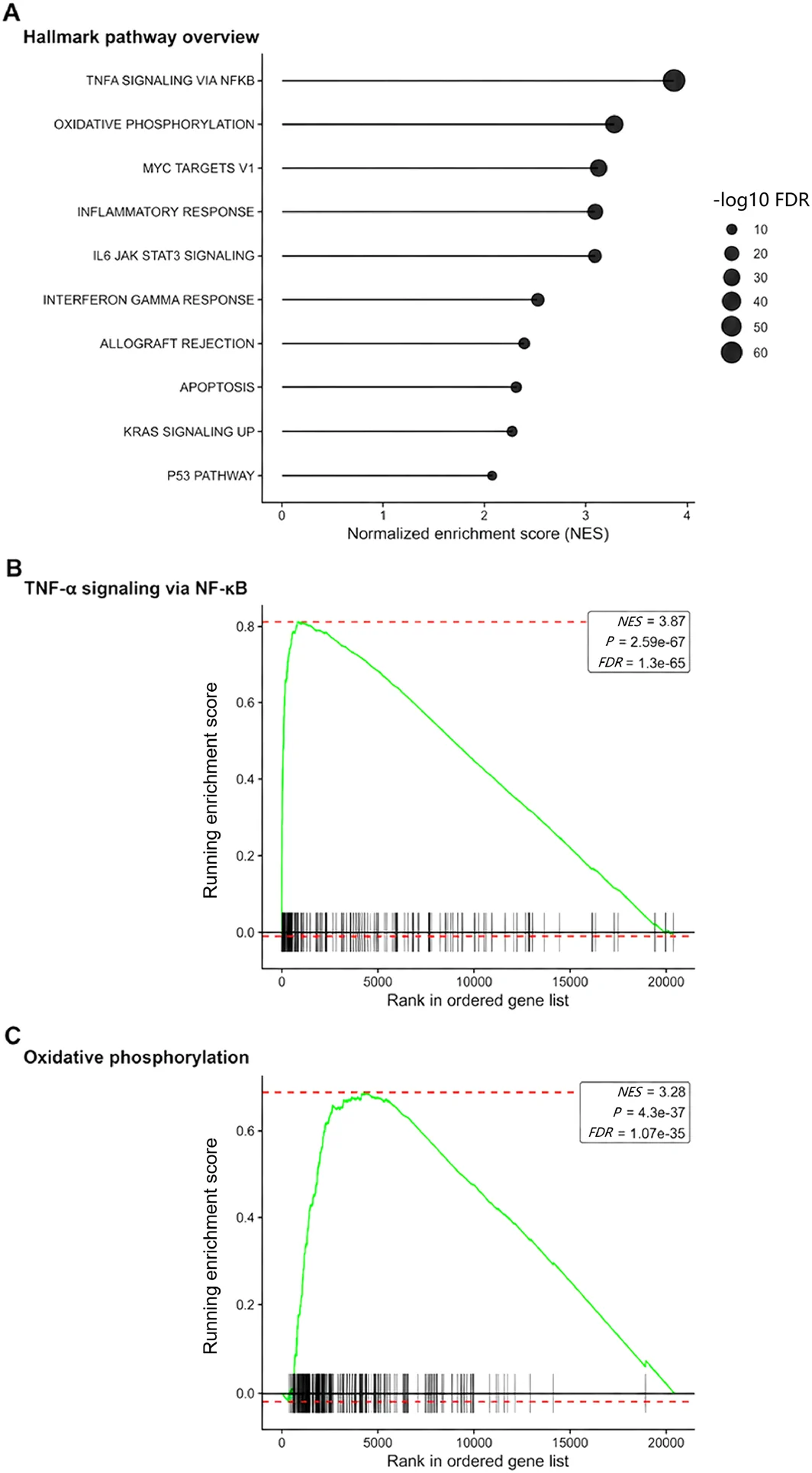
Exploratory pathway enrichment analysis of HDM‑induced epithelial stress responses. (A) Summary of selected pathway enrichment patterns in HDM‑stimulated airway epithelial cells based on exploratory gene ranking analysis, depicting representative inflammatory and metabolic pathways. (B) Enrichment pattern of genes associated with the TNF‐α signaling pathway via NF‑κB. (C) Enrichment pattern of genes associated with oxidative phosphorylation. All enrichment analyses were performed using preranked GSEA with descriptive log_2_ fold changes in an exploratory capacity and should be interpreted with caution. No formal statistical inference was applied.

As an exploratory adjunct to our experimental findings, we examined publicly available transcriptomic data (GSE243680) from HDM‐stimulated BEAS‐2B cells. Given the limited number of control replicates, this analysis was descriptive in nature and intended to generate hypotheses rather than to provide definitive mechanistic proof. Visualization of global transcriptional shifts revealed distinct expression patterns between HDM‐treated and control samples (Figure 4). Representative epithelial response genes showed heterogeneous changes, with marked upregulation of *IL‐1α* and *CXCL8*, modest increases in *JUN* and *TSLP*, and lower expression of *EGR1* and *FOS*; *IL33* was expressed at very low levels and was not retained after expression filtering. Hallmark gene set enrichment analysis (GSEA), based on the descriptive log_2_ fold‐change ranking, showed strong positive enrichment of TNF‐α signaling via NF‐κB (NES = 3.87, nominal *P* = 2.59 × 10^−^⁶⁷, FDR‐adjusted *P* = 1.30 × 10^−^⁶⁵) and oxidative phosphorylation (NES = 3.28, nominal *P* = 4.30 × 10^−^³⁷, FDR‐adjusted *P* = 1.07 × 10^−^³⁵) (Figure 5). These descriptive observations are consistent with the notion that allergen‐induced IL‐33 release as a representative indicator of epithelial activation may encompass a coordinated stress response involving both inflammatory and metabolic adaptations.

## 4 Discussion

In this study, we observed that HDM stimulation was accompanied by heightened *EGR1* expression, autophagy‐associated alterations, and amplified IL‐33 release in airway epithelial cells. Gain‐ and loss‐of‐function manipulations further demonstrated that changes in *EGR1* expression were mirrored by corresponding shifts in LC3B, p62, and PI3K‐III and by parallel changes in IL‐33 release. Moreover, HDM‐challenged mice exhibited colocalization of EGR1 and LC3B in lung tissue sections, lending in vivo credence to this response pattern. Collectively, these results support an association between *EGR1* and autophagy‐related processes in airway epithelial cells and suggest that *EGR1* may contribute to IL‐33 release as a representative indicator of epithelial activation during allergic provocation. Although the release of IL‐33 was utilized in this study as a prototypical functional readout of epithelial activation, the primary focus of this work is on the upstream *EGR1*–autophagy interface rather than the specific downstream cytokine repertoire.

Previous investigations have firmly established that EGR1 can directly govern the transcription of certain epithelial‐derived cytokines [20]. In contrast, our current findings raise the possibility that EGR1 may additionally interface with IL‐33 release as a representative indicator of epithelial activation through autophagy‐associated mechanisms. This implies that multiple regulatory strata may converge to shape epithelial inflammatory output after allergen encounter.

The airway epithelium is increasingly appreciated as an active participant in asthma pathogenesis rather than a passive physical partition [3, 4, 5]. Upon allergen inhalation, epithelial cells swiftly discharge alarmins such as IL‐33, which amplify downstream Type 2 immune responses and help initiate allergic airway inflammation [6, 7, 8]. Although the central role of IL‐33 in asthma is well recognized, the intracellular processes that control its epithelial release are still incompletely defined. Our data support the prospect that autophagy‐related activities may be involved at this step. In our experimental systems, HDM exposure was linked to elevated LC3B‐II, diminished p62, enhanced LC3B puncta formation, and increased IL‐33 release, whereas pharmacological blockade of autophagy curtailed IL‐33 release. These observations align with the view that, under these conditions, autophagy‐related activity may contribute to shaping epithelial inflammatory output beyond its canonical homeostatic degradative role [11, 12, 13].

A salient observation emerging from this study is the consistent linkage of EGR1 to these autophagy‐associated changes. EGR1 functions as a rapid stress‐responsive transcription factor induced by inflammatory and environmental cues and has previously been implicated in airway IL‐33 release as a representative indicator of epithelial activation and Type 2 inflammation [17, 18, 19]. Earlier work had already posited a connection between *EGR1* and epithelial *IL‐33* expression [20]. This study extends this body of evidence by showing that modulation of *EGR1* expression is accompanied by corresponding adjustments in LC3B, p62, PI3K‐III, and IL‐33 release as representative indicators of epithelial activation. Although this does not establish direct transcriptional control of the autophagy machinery by EGR1, it does support a model in which EGR1 may act upstream of autophagy‐related processes that are coupled to epithelial alarmin release.

Our exploratory transcriptomic analysis provides additional contextual framing for these experimental observations, albeit with important caveats. The publicly accessible airway epithelial dataset showed heterogeneous changes in immediate‐early response genes and epithelial alarmins, together with marked induction of inflammatory mediators and strong enrichment of TNF‐α signaling via NF‐κB. These inflammatory signaling networks may converge on NF‐κB, a central transcriptional regulator that integrates cytokine and stress signals during inflammatory responses [21, 22]. These findings provide contextual evidence that the *EGR1*‐associated response documented in our wet‐laboratory experiments occurs within a broader epithelial stress program evoked by allergen exposure, but do not independently corroborate EGR1 induction. However, given the descriptive nature of the gene‑level analysis and the absence of formal gene‑level statistical testing, these transcriptomic findings should be regarded strictly as hypothesis‐generating rather than direct mechanistic corroboration.

We also noted suggestive enrichment of oxidative phosphorylation‐related pathways in the exploratory transcriptomic analysis. This raises the possibility that IL‐33 release as a representative indicator of epithelial activation under allergen duress may be accompanied by metabolic adaptation. Accumulating evidence indicates that inflammatory activation and metabolic status are intimately coupled in numerous cell types, including epithelial and immune cells [23, 24, 25, 26, 27, 28]. Nevertheless, we did not directly quantify mitochondrial respiration, reactive oxygen species generation, or metabolic fluxes and thus cannot conclude that metabolic reprogramming serves as a mechanistic driver of the *EGR1*–autophagy axis. These preliminary observations merely suggest that allergen‐induced epithelial stress may encompass coordinated inflammatory and metabolic adjustments that warrant dedicated investigation in future studies.

**FIGURE 6:**
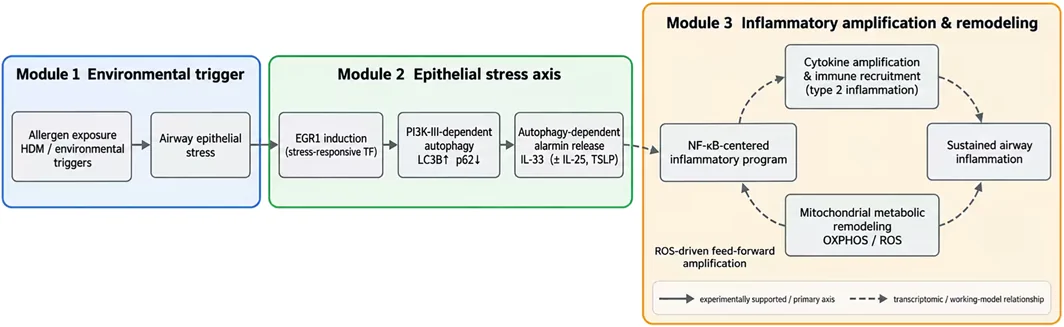
Proposed working model of early growth indicator 1 (EGR1)‐associated autophagy‐related epithelial stress responses in allergic airway inflammation. Environmental allergen exposure (e.g., house dust mite, HDM) induces epithelial stress responses, leading to increased EGR1 expression in airway epithelial cells. EGR1 upregulation is accompanied by PI3K‐III‐associated autophagy‐related changes, which may contribute to the facilitation of epithelial alarmin release (e.g., IL‐33). This process likely occurs within a broader epithelial stress response that may involve inflammatory signaling and metabolic adaptation. Solid arrows indicate associations supported by experimental observations in this study; dashed arrows represent potential links requiring further mechanistic validation. Created with BioRender.com.

Taken together, our results support a working model in which HDM exposure induces *EGR1* expression in airway epithelial cells, which is accompanied by PI3K‐III‐associated autophagy changes and heightened IL‐33 release as a representative indicator of epithelial activation, as reflected by augmented IL‐33 release (Figure 6). This conceptual framework is summarized in Figure 6. Within this schema, EGR1 may function as an upstream stress‐responsive regulator, whereas autophagy‐related processes may facilitate the intracellular conditions that permit epithelial alarmin release. This model does not exclude the participation of alternative pathways—including transcriptional and inflammatory signaling networks—in the regulation of epithelial‐derived cytokines. Rather, it highlights autophagy‐related activity as a plausible additional tier of control in epithelial inflammatory activation.

### 4.1 Limitations

Several limitations of this study merit explicit acknowledgment. First, direct autophagic flux assays employing lysosomal inhibitors (e.g., chloroquine) were not performed; consequently, we cannot definitively distinguish between enhanced autophagic degradation and impaired lysosomal clearance. The term “autophagy‐related changes” is therefore employed throughout to reflect this uncertainty. Second, although IL‐33 release was measured as a functional correlate of autophagic activity, we did not delineate the precise trafficking mechanisms linking autophagosomes to IL‐33 secretion. Future studies will be required to establish whether the autophagy machinery directly participates in sorting IL‐33 or merely creates a permissive cellular state for alarmin release. Third, the pharmacological inhibitor 3‐MA was used at a relatively high concentration (10 mmol/L), which may exert off‐target effects beyond Class III PI3K inhibition; orthogonal approaches such as Vps34‐specific inhibitors or genetic knockdown were not employed. Fourth, the in vivo experiments utilized a small sample size (*n* = 4 per group), which may limit statistical power, and immunofluorescence colocalization was not validated with epithelial‐specific markers, precluding definitive assignment of the observed signals to airway epithelial cells. Fifth, the transcriptomic analysis was exploratory and descriptive at the gene level, lacking formal gene‑level statistical inference; thus, the pathway‑level GSEA findings should be interpreted with caution and require validation in future studies. Finally, our functional readout was restricted primarily to IL‐33; other epithelial alarmins such as TSLP and IL‐25 may likewise be influenced by related processes and warrant further investigation.

In conclusion, our findings substantiate an association between EGR1, PI3K‐III‐related autophagy alterations, and IL‐33 release as a representative indicator of epithelial activation during allergic stimulation, as reflected by enhanced IL‐33 release. This work contributes an integrated perspective on how airway epithelial cells respond to allergen exposure during the incipient phases of allergic airway inflammation. Further investigations employing more direct mechanistic and genetic approaches are required to delineate precisely how EGR1 interfaces with autophagy‐related pathways and how these processes collectively regulate epithelial inflammatory output in asthma.

## 5 Conclusion

In summary, this study delineates a consistent association between *EGR1* expression, autophagy‐related alterations, and IL‐33 release as a representative indicator of epithelial activation in the context of allergen challenge, with IL‐33 release serving as a representative functional readout. Utilizing both in vitro and in vivo models of HDM‐induced allergic airway inflammation, we demonstrate that manipulation of *EGR1* is paralleled by coordinated changes in LC3B, p62, PI3K‐III, and epithelial inflammatory output, thereby supporting a nexus between epithelial stress responses and autophagy‐related processes.

Our findings suggest a model wherein EGR1 may serve as an upstream stress‐responsive regulator linked to PI3K‐III‐associated autophagy processes, which, in turn, may facilitate epithelial alarmin release. This work expands current understanding of airway IL‐33 release as a representative indicator of epithelial activation by underscoring autophagy‐related activity as a potential supplementary layer of regulation in early allergic inflammation.

Further studies are warranted to unravel the molecular mechanisms underlying the interplay between EGR1 and autophagy‐related pathways and to ascertain how these processes modulate epithelial‐derived cytokines in asthma.

## Author Contributions

**Xinyang Wang:** investigation, formal analysis, writing – original draft. **Jie Ren:** investigation, data curation. **Yan Zhao:** methodology, validation, writing – review and editing. **Zhengxiu Luo:** conceptualization, supervision, funding acquisition. All authors have read and agreed to the published version of the manuscript.

## Funding

The authors have nothing to report.

## Conflicts of Interest

The authors declare no conflicts of interest.

## Ethics Statement

All animal procedures were approved by the Institutional Animal Care and Use Committee, Children's Hospital of Chongqing Medical University (Approval No. CHCMU‐IACUC20230804005).

## Data Availability

The datasets generated and/or analyzed during this study are available from the corresponding author upon reasonable request. Public transcriptomic data analyzed in this study are available from the GEO database under accession number GSE243680.
